# Identification of the Endogenous Key Substrates of the Human Organic Cation Transporter OCT2 and Their Implication in Function of Dopaminergic Neurons

**DOI:** 10.1371/journal.pone.0000385

**Published:** 2007-04-25

**Authors:** Dirk Taubert, Gundula Grimberg, Werner Stenzel, Edgar Schömig

**Affiliations:** 1 Department of Pharmacology, Medical Hospital of the University of Cologne, Cologne, Germany; 2 Division of Neuropathology, Medical Hospital of the University of Cologne, Cologne, Germany; University of Washington, United States of America

## Abstract

**Background:**

The etiology of neurodegenerative disorders, such as the accelerated loss of dopaminergic neurons in Parkinson's disease, is unclear. Current hypotheses suggest an abnormal function of the neuronal sodium-dependent dopamine transporter DAT to contribute to cell death in the dopaminergic system, but it has not been investigated whether sodium-independent amine transporters are implicated in the pathogenesis of Parkinson's disease.

**Methodology/Principal Findings:**

By the use of a novel tandem-mass spectrometry-based substrate search technique, we have shown that the dopaminergic neuromodulators histidyl-proline diketopiperazine (cyclo(his-pro)) and salsolinol were the endogenous key substrates of the sodium-independent organic cation transporter OCT2. Quantitative real-time mRNA expression analysis revealed that OCT2 in contrast to its related transporters was preferentially expressed in the dopaminergic regions of the substantia nigra where it colocalized with DAT and tyrosine hydroxylase. By assessing cell viability with the MTT reduction assay, we found that salsolinol exhibited a selective toxicity toward OCT2-expressing cells that was prevented by cyclo(his-pro). A frequent genetic variant of OCT2 with the amino acid substitution R400C reduced the transport efficiency for the cytoprotective cyclo(his-pro) and thereby increased the susceptibility to salsolinol-induced cell death.

**Conclusions/Significance:**

Our findings indicate that the OCT2-regulated interplay between cyclo(his-pro) and salsolinol is crucial for nigral cell integrity and that a shift in transport efficiency may impact the risk of Parkinson's disease.

## Introduction

Membrane transporters are essential for specificity and normal function of a cell. Abnormalities in the function of neuronal sodium-dependent monoamine transporters, such as the dopamine transporter DAT, have long been implicated in the etiology of a variety of neurodegenerative disorders including Parkinson's disease (PD).[1] However, the physiological and pathological roles of other amine transporters in brain, such as the organic cation transporter OCT2, are still unknown.

OCT2, which is encoded by the solute carrier (SLC) 22A2 gene, is known to mediate sodium-independent electrogenic transport of positively charged organic compounds of a wide range of different molecular structures having sizes up to 350 Da.[2] It is therefore classified as polyspecific transporter along with the organic cation transporter OCT1 (SLC22A1) and the extraneuronal monoamine transporter EMT (SLC22A3) that comprise a group of closely related transporters with more than 50% amino acid sequence identity localized to the chromosomal site 6q26. Also, OCT2 is regarded to share extensively overlapping substrate specificities with OCT1 and EMT.[2]

Based upon findings of high OCT2 expression in kidney and OCT2-mediated translocation of cationic drugs and synthetic model compounds like tetraethylammonium (TEA), current hypotheses suggest that OCT2 is centrally involved in the renal clearance of diverse cationic xenobiotics.[3] However, this assumption about the functional role of OCT2 derives from limited information. Expression of OCT2 has been assayed only in a small number of tissues. In particular, functional implication of the neuronal OCT2 localization reported[4], [5] has not been sufficiently explored. Moreover, to date, systematic investigations to elucidate the endogenous substrates of OCT2 have not been performed. In particular, low affinities and transport efficiencies that have been observed for currently proposed biogenic OCT2 substrates – such as the catecholamines, serotonin, histamine or tyramine[6] – suggest that the endogenous key substrates of OCT2 are still unidentified.

## Results and Discussion

### A novel mass spectrometry based method for systematic identification of substrates for cell membrane transporters

The basic design to study the substrates of transporters is to determine the uptake of a selected test substance into a cell line that expresses the transporter of interest in comparison to the same cell line lacking the transporter. The difference in intracellular accumulation of the substance (which can be measured in the cell lysates [7]) then reflects the specific transporter-mediated uptake. To reliably identify the leading endogenous substrates of a transporter this approach requires sequential testing of large numbers of potential substrates in separate uptake experiments, which is at best inefficient but often impracticable (e.g. due to limited availability of authentic standards). To overcome this limitation we developed a technique that allows the systematic identification of the preferred endogenous substrates of a transporter in a single uptake experiment by employing complex matrices derived from blood or other organs containing the complete, but a priori unknown spectrum of possible substrates. The compounds with the highest cellular accumulation consequently represent the best endogenous substrates. Analysis of these compounds is performed in the cell lysates using liquid chromatography (LC) tandem mass spectrometry (MS-MS) by repetition of a single ion monitoring (SIM) algorithm for the mass range (m/z) in which the possible substrates are expected to fall, as described in ‘Materials and Methods’. Compared with full scan approaches that utilize only the first MS dimension for detection, the use of the two-dimension MS considerably raises sensitivity achieving detection limits in the low nanomolar concentration range, and provides simultaneously the unique fragment ion masses to a specific parent mass, which in most cases allow the assignment of a structure to the substance.

Identification of a substrate with this method essentially depends on the presence of the compound in the incubation matrix. Therefore, we performed the uptake experiments by employing fetal bovine serum as a concentrated mixture of a broad spectrum of all endogenous compounds derived from the total organism. To specifically address the role of OCT2 in the CNS an additional set of uptake experiments was conducted with whole brain extract from adult rats.

### Identification and kinetic characterization of histidyl-proline diketopiperazine and salsolinol as endogenous key substrates of OCT2

**Figure 1:**
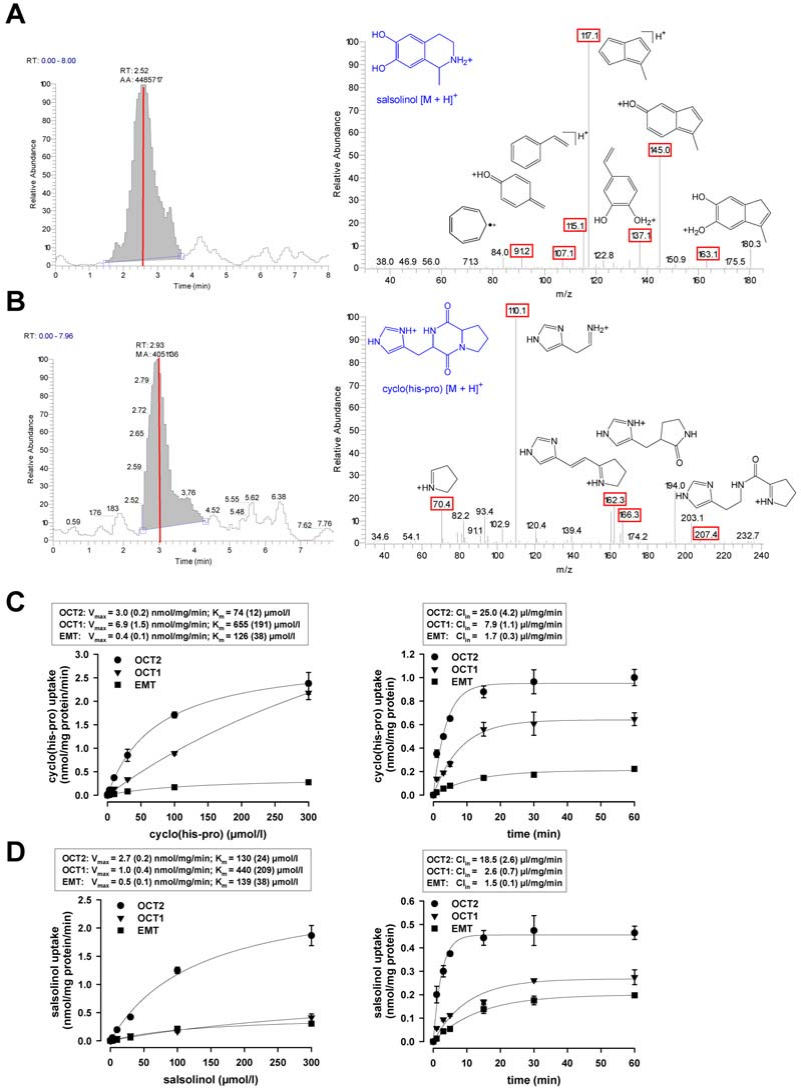
Identification and transport kinetic characterization of cyclo(his-pro) and salsolinol as endogenous substrates of the organic cation transporter OCT2. (A and B) Chromatograms and corresponding spectra of ESI-MS-MS fragmentation of the molecular ions [M+H]^+^ at m/z 180 and 235 derived from lysates of OCT2-transfected HEK-293 cells versus empty vector transfected HEK-293 cells after 30 min of incubation with 100% fetal bovine serum (pH 7.4, 37°C) following 60 min preincubation in buffer solution. Specific fragment masses are indicated in red and assigned to the proposed fragment ion structures; the parent ions are shown in blue. (C and D) Concentration dependence, maximal transport rate V_max_ (mean (s.e.m.)) and Michaelis-Menten constant K_m_ (mean (s.e.m.)) of specific uptake of cyclo(his-pro) or salsolinol into HEK-293 cells transfected with OCT2 or the related cation transporters OCT1 and EMT after 1 min of loading (left panel); time dependence and influx clearance Cl_in_ (mean (s.e.m.)) of specific uptake after incubation with 10 µmol/l of cyclo(his-pro) or salsolinol (right panel) (n = 3 independent experiments each). N-methylation of intracellular salsolinol was negligible (maximal 2.5% of salsolinol concentration after 60 min of incubation).

Uptake experiments were conducted in OCT2-transfected human embryonic kidney cells (HEK-293) by incubation with fetal bovine serum. Specific OCT2-mediated uptake of a compound from serum was assessed in comparison to HEK-293 cells without OCT2 expression by running positive electrospray ionization tandem mass spectrometry analysis (ESI-LC-MS-MS). Significant specific uptake (>2-fold above the control) was solely observed for the molecular ions [M+H]^+^ at mass-to-charge ratios m/z of 180 and 235, as indicated by the difference chromatograms obtained from the SIM experiments (**Fig. 1A**). The corresponding collision-induced dissociation (CID) spectra could be assigned to salsolinol and histidyl-proline diketopiperazine (cyclo(his-pro)), which was confirmed by product ion spectra of the authentic standards (**Fig. 1A**). This result was further confirmed by running the LC-MS-MS algorithm with whole brain extract from adult rats as a source of potential endogenous substrates (**Fig. S1**). Moreover, the presence of significant concentrations of cyclo(his-pro) and salsolinol in fetal bovine serum (FBS) and brain extract was confirmed by LC-MS-MS selected ion and selected reaction monitoring (FBS: 287 nmol/l cyclo(his-pro) and 4.4 nmol/l salsolinol; brain extract: 132 nmol/l cyclo(his-pro) and 3.8 nmol/l salsolinol).

**Figure 2:**
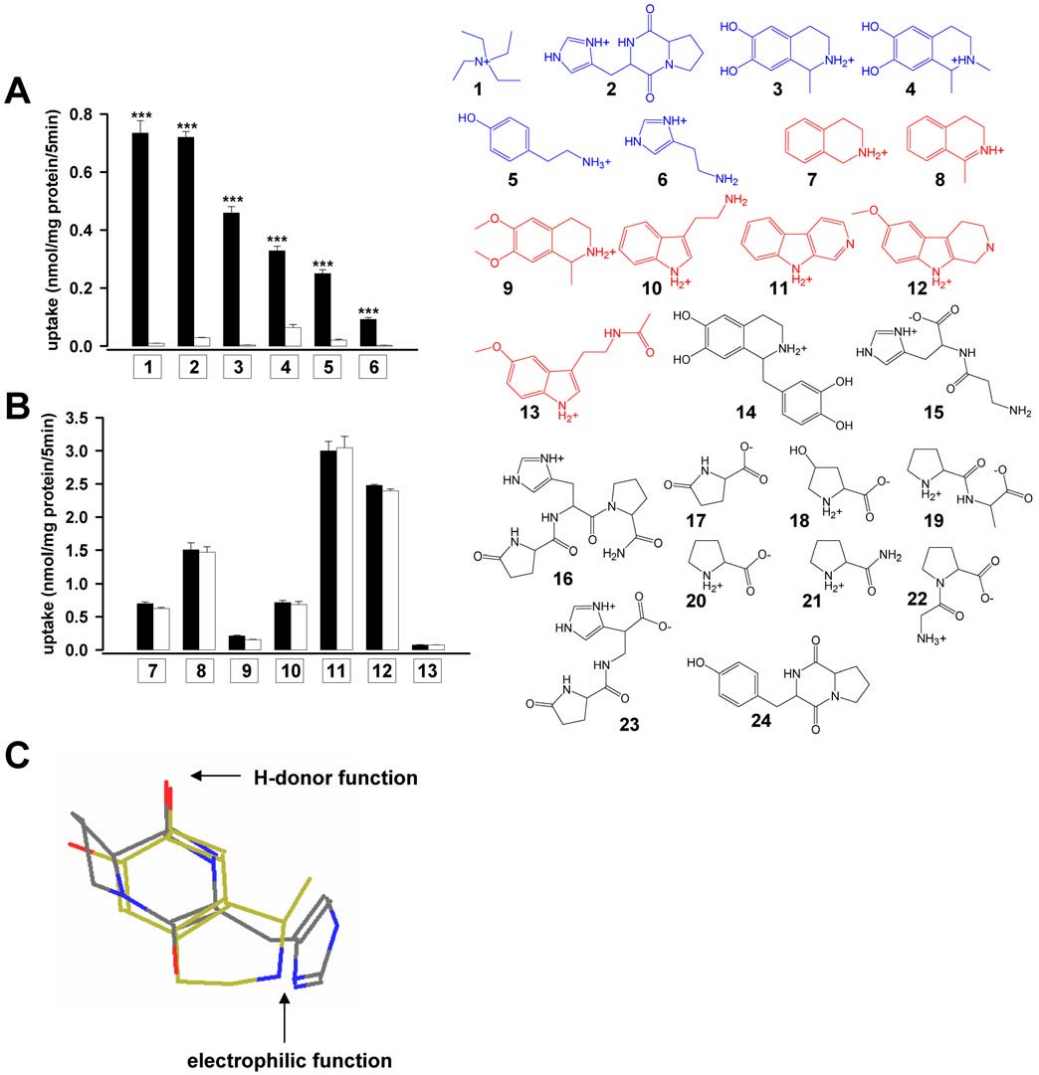
Selectivity of cyclo(his-pro) and salsolinol as endogenous substrates of the organic cation transporter OCT2. (A and B) Accumulation of selected compounds in HEK-293 cells expressing OCT2 (black bars) compared to cells expressing empty vector (open bars) after 5 min of incubation with 10 µmol/l each (n = 3, mean±s.e.m.; ***P<0.001). Substances showing significantly higher accumulation in OCT2 than in control cells (specific OCT2-mediated transport) are depicted in blue; substances with equal accumulation in OCT2 and control cells (unspecific OCT2-independent transport) are shown in red, and substances without detectable uptake in OCT2 or control cells are shown in black. (1: tertaethylammonium; 2: cyclo(his-pro); 3: salsolinol; 4: N-methyl-salsolinol; 5: tyramine; 6: histamine; 7: 1,2,3,4-tetrahydroisoquinoline; 8: 1-methyl-3,4-dihydroisoquinoline; 9: salsolidine; 10: tryptamine; 11: norharmane; 12: 6-methoxy-1,2,3,4-tetrahydro-β-carboline; 13: melatonin; 14: tetrahydropapaveroline; 15: carnosine; 16: thyrotropin-releasing hormone (TRH); 17: pyroglutamate; 18: 4-hydroxy-proline; 19: prolylalanine; 20: proline; 21: prolineamide; 22: glycylproline; 23: pyroglutamyl-histidine; 24: cyclo(tyr-pro)). (C) The overlay of energy minimized conformations (MM2 computation, Chem3D Pro software) of native cylo(L-his-L-pro) (enol tautomer, endo-conformation)[24] and R-salsolinol uncovers close structural similarities, suggesting a planar ring system, an electrophilic center, and a nucleophilic moiety with H-donor properties at a constant distance (of 6.5±0.3 Å) as key properties of a OCT2-specifc substrate.

Analysis of the transport kinetics of cyclo(his-pro) and salsolinol uptake in OCT2-expressing HEK-293 cells revealed additional evidence to suggest these compounds as specific endogenous key substrates of OCT2:

1. The efficiency of transport (V_max_ /K_m_) was higher than for other endogenous compounds, including histamine and tyramine, which were so far suggested to be the best endogenous substrates of OCT2 (**Fig. S2**).[6] By comparison, the catecholamine neurotransmitters (in particular the salsolinol precursor dopamine) exhibited no significant OCT2-mediated transport.[8]
2. The transport efficiency approximated the high level of the unspecific OCT2 substrate TEA (**Fig. 2A** **; Fig. S2**).
3. HEK-293 cells expressing the isoform transporters OCT1 and EMT revealed considerably lower efficiency and clearance for transport of cyclo(his-pro) and salsolinol (**Fig. 1, C and D**).
4. Precursors, metabolites, and endogenous compounds structurally related to cyclo(his-pro) and salsolinol failed to show specific OCT2-mediated transport, except from N-methyl-salsolinol that revealed about two-thirds of the salsolinol uptake rate (**Fig. 2, A and B**).
5. Cyclo(his-pro) and salsolinol share common structural features that appear to be essential for a specific OCT2 substrate (**Fig. 2C**). In agreement with the reported characteristics of OCT2-mediated transport, uptake of cyclo(his-pro) and salsolinol was found to be electrogenic in translocating only the positively charged molecule (**Fig. S3**) and independent of the transmembraneous sodium gradient (**Fig. S4**).

### Profiling of OCT2, cyclo(his-pro) and salsolinol in dopaminergic brain areas

**Figure 3:**
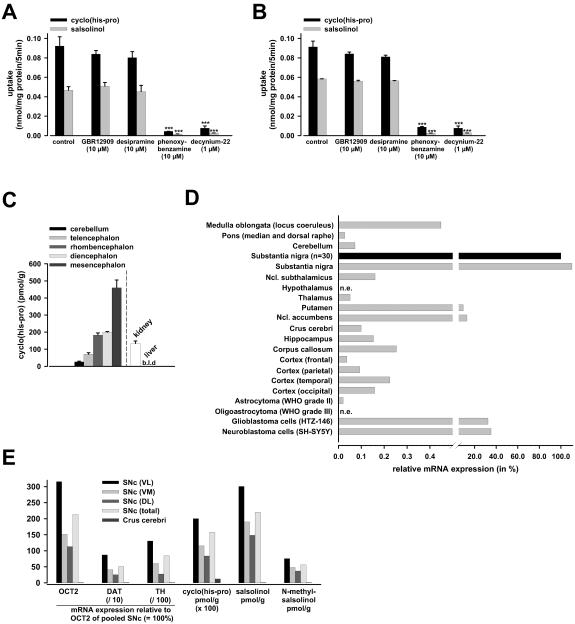
OCT2, cyclo(his-pro), and salsolinol in brain. Uptake of cyclo(his-pro) and salsolinol (10 µmol/l, 5 min incubation) into human neuroblastoma (SH-SY5Y) cells (A) and human glioblastoma cells (HTZ-146) (B) was not different from control conditions when coincubated with selective inhibitors of dopamine (GBR12909, 10 µmol/l) or norepinephrine (desipramine, 10 µmol/l) transport, but was almost abolished after specific inhibition of organic cation transport via OCT1, OCT2, and EMT with phenoxybenzamine (10 µmol/l) or decynium-22 (1 µmol/l)[25] (n = 3, mean±s.e.m.; ***P<0.001). OCT2 expression was similar in SH-SY5Y and HTZ-146 cells showing 35% and 32% of the level in substantia nigra respectively. Levels of mRNA expression of DAT, NET, OCT1, and EMT in SH-SY5Y cells were 4.63%, 1.64%, 0.04%, and 0%, and in HTZ-146 cells 0%, 0.05%, 0.70%, and 0.37% of the respective OCT2 expression ( = 100%). Hence, neither the neuronal catecholamine transporters nor the isotypic cation transporters OCT1 and EMT can account for a significant fraction of total uptake of cyclo(his-pro) and salsolinol in brain cells. (C) Concentration of cyclo(his-pro) in rat brain divisions was highest in mesencephalon encompassing the dopaminergic region substantia nigra. In comparison, in rat kidney (with the highest OCT2 expression)[15], cyclo(his-pro) concentration was lower, and in liver (lacking OCT2)[15], no cyclo(his-pro) was detected (n = 6, mean±s.e.m.; levels were not different between male (3) and female (3) rats; b.l.d.: below limit of detection). (D) Relative quantitative real-time expression of OCT2 mRNA in distinct areas of normal human brain and neuroepithelial tumors (TaqMan Assay) normalized to GAPDH. Expression was highest in substantia nigra pars compacta (SNc) and is given relative to an equally pooled sample derived from 30 normal donors aged 23–68 years (relative expression = 100%; black bar). Specimens of other normal brain areas were derived from a male donor aged 63 years; n.e. indicates no expression of OCT2. The expression profile of the organic cation transporters and DAT in SNc with 0.048% (OCT1), 0.023% (EMT), and 3.66% (DAT) respectively of the mRNA level of OCT2 ( = 100%) resembled that of SH-SY5Y cells which therefore represent an adequate model of OCT- and DAT-mediated transport in SNc. (E) Correlation of OCT2, DAT, and tyrosine hydroxylase (TH) mRNA expression (relative to OCT2 in pooled SNc ( = 100%)) with intracellular concentrations of cyclo(his-pro), salsolinol and N-methylsalsolinol in microdissection preparations of total SNc, the SNc adjacent crus cerebri (right section), and ventrolateral (VL), ventromedial (VM), and dorsolateral (DL) regions of SNc (left section) derived from a female donor aged 70 years. The neuron-specific expression profile of DAT and TH reflects the characteristic differences in the loss of dopaminergic neurons in SNc regions with normal aging.[26]

The specific biological roles of cyclo(his-pro) and salsolinol in human physiology and pathophysiology are largely unknown. On the basis of experimental and animal studies, it is hypothesized that both compounds function as endogenous neuromodulators of the central dopaminergic transmission.[9], [10], [11], [12] Cyclo(his-pro) is supposed to increase and salsolinol to decrease neuronal sensitivity to dopamine. These effects were shown to take place without direct interaction with dopamine receptors or changes in neuronal excitability in the absence of dopamine.[9], [10], [11], [13] In agreement with the lacking affinity to catecholamine receptors, pharmacological characterization of transport into human dopaminergic neuroblastoma (SH-SY5Y) and primary human glioblastoma (HTZ-146) cells revealed that cyclo(his-pro) and salsolinol were not translocated by the neuronal dopamine transporter (DAT) or the neuronal norepinephrine transporter (NET); in addition, near total abolishment of uptake in the presence of specific inhibitors of the organic cation transporters indicated that no further transporters were involved (**Fig. 3, A and B**). The failure of DAT to translocate cyclo(his-pro) or salsolinol is also in keeping with previous reports.[9], [10], [11], [13] Further evidence for the failing contribution of NET in cyclo(his-pro) or salsolinol transport was obtained from uptake experiments in NET-transfected HEK-293 cells (**Fig. S5**).

Cyclo(his-pro) was initially discovered as a stable hypothalamic metabolite of the thyrotropin-releasing hormone (TRH), but is also synthesized de novo endogenously in the brain;[9] analysis of the cyclo(his-pro) distribution in rat brain likewise indicates its prevalence in dopaminergic areas (**Fig. 3C**). Salsolinol arises from enzymatic condensation of dopamine with either acetaldehyde or pyruvic acid;[14] hence, highest concentrations were detected in dopaminergic brain areas including substantia nigra.[14] Intriguingly, OCT2 was found to be preferentially expressed in the dopaminergic brain regions with the highest central expression in substantia nigra pars compacta (SNc), the area with the highest density of dopamine cell bodies in the CNS (**Fig. 3D**). This finding agrees with a previous expression study of our group in rat brain.[15] The demonstration of significant levels of OCT2 in SH-SY5Y cells (**Fig. 3D**) and also in primary human dopaminergic neuronal cells[5] further supports a prominent role of OCT2 in the central dopaminergic system. In comparison with OCT2, dopaminergic expression of OCT1 and EMT was negligible with only 0.048% and 0.023%, respectively in human SNc, and 0.040% and 0%, respectively in SH-SY5Y cells. Analysis of SNc subregions in human brain revealed a strong correlation of OCT2 with the neuron-specific expression of DAT and tyrosine hydroxylase (TH) along with the intracellular levels of the endogenous OCT2 substrates. In contrast, the SNc adjacent area of the crus cerebri lacked significant OCT2 expression; consequently, intracellular concentrations of cyclo(his-pro), salsolinol and N-methylsalsolinol were negligible (**Fig. 3E**). In comparison to OCT2 expression in the brain, OCT2 levels in peripheral tissues were all considerably lower than in SNc, apart from kidney where OCT2 may be involved in the clearance of cyclo(his-pro) (**Fig. 3C** **; Fig. S6**). These data *further support the notion of a highly specific function for OCT2 in nigral dopaminergic system.

### Impact of OCT2 on neurodegeneration and neuroprotection

**Figure 4:**
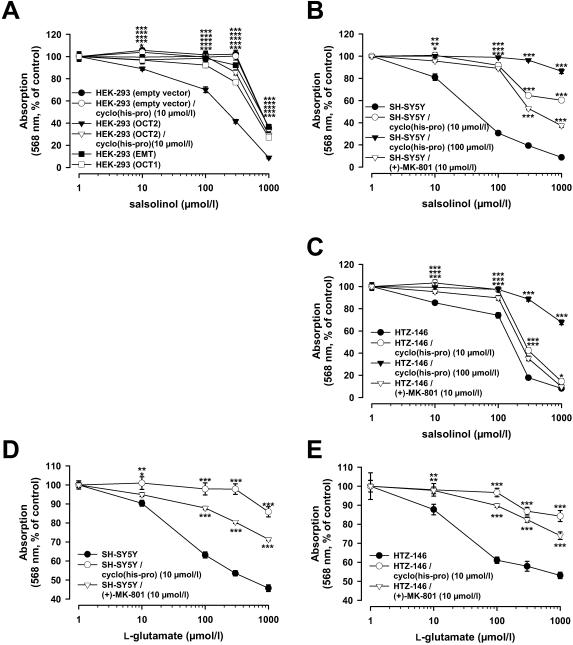
Selective cytotoxic effects of salsolinol and cytoprotective effects of cyclo(his-pro) toward OCT2-expressing cells. Cell viability was assessed by MTT reduction assay; resulting formazane formation was determined by absorbance at 568 nm. (A) After 48 h exposure to different concentrations of salsolinol, HEK-293 cells expressing OCT2 showed higher cell loss than HEK-293 cells expressing empty vector, OCT1 or EMT. The respective EC_50_ values (95% confidence intervals) for salsolinol were 192 (174 to 212), 1112 (900 to 1375), 627 (525 to 748), and 998 (780 to 1277) µmol/l. 12 h of incubation with cyclo(his-pro) (10 µmol/l) prior to salsolinol treatment prevented degeneration of OCT2-transfected HEK-293 cells (EC_50_ = 763 (655 to 889) µmol/l), but had no effect on empty vector transfected HEK-293 cells (EC_50_ = 1073 (870 to 1324) µmol/l) (n = 24–48; mean±s.e.m.; *P<0.05, ***P<0.001). (B and C) Cytotoxicity of salsolinol and its protection by pretreatment with cyclo(his-pro) (10 and 100 µmol/l) were confirmed in endogenously OCT2-expressing SH-SY5Y and HTZ-146 cells. The EC_50_ values in non-pretreated, and for 1 h with 10 µmol/l and 100 µmol/l cyclo(his-pro) pretreated SH-SY5Y cells were 49 (44 to 56), 1054 (888 to 1251), and 6568 (5313 to 8119) µmol/l of salsolinol, and the respective values in HTZ-146 cells were 138 (120 to 160), 318 (268 to 377), and 2176 (1727 to 2741) µmol/l of salsolinol. (D and E) Cyclo(his-pro) prevented SH-SY5Y and HTZ-146 cells from glutamate-induced cytotoxicity. EC_50_ values for glutamate were 389 (323 to 467) vs. 6461 (4132 to 10102) µmol/l in SH-SY5Y cells, and 585 (490 to 699) vs. 4768 (3660 to 6211) when comparing non-pretreated cells with cells pre-treated for 1 h with 10 µmol/l cyclo(his-pro). Cell death caused by glutamate (D and E) as well as salsolinol (B and C) was also attenuated in the presence of a specific inhibitor of NMDA receptor-operated calcium channels ((+)-MK-801, 10 µmol/l), indicating that blockage of excitotoxic calcium entry may contribute to the protective mechanism of cyclo(his-pro). The EC_50_ values for glutamate were 1986 (1744 to 2261) in SH-SY5Y and 2316 (1723 to 3112) µmol/l in HTZ-146 cells, and the EC_50_ for salsolinol was 475 (430 to 524) in SH-SY5Y and 242 (202 to 291) µmol/l in HTZ-146 cells (n = 16–32; mean±s.e.m.; *P<0.05, **P<0.01, ***P<0.001).

Beyond its physiological neuromodulatory function, there is considerable interest in the pathophysiologic role of salsolinol. In patients with idiopathic Parkinson's disease (PD) elevated brain levels of salsolinol and its N-methylated derivatives were observed.[14], [16], [17] It has been hypothesized that especially N-methyl-salsolinol is etiologically involved in apoptotic nigral cell death in PD by promoting calcium-triggered disruption of mitochondrial permeability transition.[14] Here, we show that salsolinol toxicity is preferentially targeted to OCT2 expressing cells. Assessing cell viability by MTT reduction assay, we found that salsolinol exposure (over 48 h) caused concentration-dependent damage of OCT2-transfected HEK-293 cells as well as of SH-SY5Y and HTZ-146 cells, which demonstrated a significant endogenous OCT2 expression (**Fig. 4, A to C**). In contrast, HEK-293 cells transfected with OCT1, EMT, or empty vector were affected only at 3–5 fold higher salsolinol concentrations than the OCT2-transfected HEK-293 cells (**Fig. 4A**). After 48 h incubation of OCT2-transfected HEK-293 cells with 10 and 100 µmol/l salsolinol, intracellular concentrations of the metabolite N-methyl-salsolinol were 0.89±0.06 and 7.56±0.41 nmol/mg protein, with SH-SY5Y cells 0.18±0.01 and 1.28±0.11 nmol/mg protein, and HTZ-146 cells 0.17±0.01 and 1.16±0.06 nmol/mg protein respectively (n = 3; mean±s.e.m.). As no unchanged salsolinol was detected, this suggests that the intracellular synthesized N-methyl derivative represents the primary active cytotoxic agent.

Synthetic dipeptides that are structurally related to cyclo(his-pro) have been reported to exert neuroprotective antiapoptotic effects and to attenuate glutamate-induced elevations of intracellular calcium.[18] Therefore, we asked whether cyclo(his-pro) could prevent OCT2-expressing cells from salsolinol-induced cell death and whether the protective effect also extends to prevention of glutamate-induced excitotoxic cell injury. Pretreatment of OCT2-transfected HEK-293 cells for 12 h, or SH-SY5Y and HTZ-146 cells for 1 h with cyclo(his-pro) prior to incubation with salsolinol substantially diminished cell degeneration (**Fig 4, A to C**). SH-SY5Y and HTZ-146 cells were also shown to express subunits (NR1, 2a and 2b) of ionotropic NMDA glutamate receptors, and consequently exhibited injury in response to glutamate (**Fig 4, D and E**); the effect could be attributed to NMDA receptor-activated Ca^2+^ influx, since glutamate toxicity was attenuated by coincubation with a selective antagonist of NMDA receptor-gated calcium channels ((+)-MK-801, 10 µmol/l). Similarly, pretreatment with cyclo(his-pro) (10 µmol/l) prevented glutamate-induced damage of HTZ-146 cells (**Fig 4, D and E**). In turn, the presence of (+)-MK-801 reduced salsolinol-induced toxicity in HTZ-146 cells (**Fig. 4, A to C**). This suggests that the protective mechanism of cyclo(his-pro) in neuronal cells implies an inhibition of excitotoxic calcium influx, thereby preventing mitochondrial impairment and subsequently apoptosis.

### Impact of genetic variants of OCT2 on transport kinetics of cyclo(his-pro) and salsolinol and cell stress response

**Figure 5:**
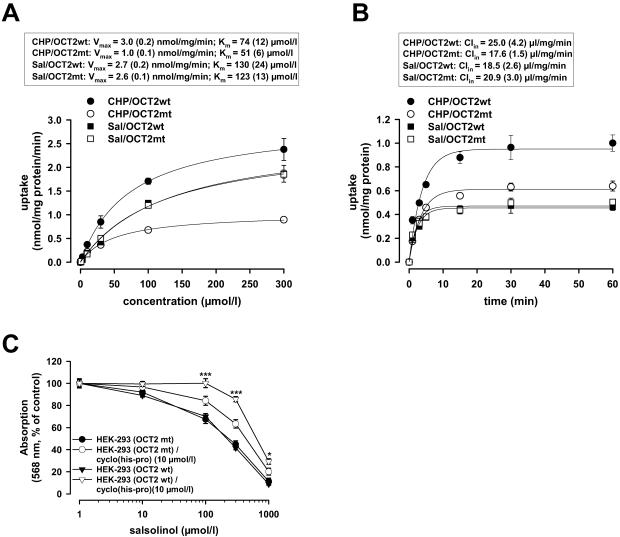
Impact of the R400C polymorphism of OCT2 on transport kinetics. **(A)** Concentration dependence, maximal transport rate V_max_, and Michaelis-Menten constant K_m_ of specific uptake of cyclo(his-pro) or salsolinol into HEK-293 cells transfected with wild-type (wt) 400R or mutant (mt) 400C variant after 1 min of loading (n = 3, mean±s.e.m). **(B)** Time dependence and influx clearance Cl_in_ of specific uptake after incubation with 10 µmol/l of cyclo(his-pro) or salsolinol (n = 3, mean±s.e.m). For cyclo(his-pro), transport efficiency (V_max_/K_m_) and clearance of 400C were lower, achieving 48% (P = 0.0017) and 70% (P = 0.002) of the 400R reference respectively. For salsolinol, transport efficiency and clearance showed no significant differences between 400R and 400C (P = 0.995 and 0.591 respectively). **(C)** Viability of HEK-293 cells expressing the OCT2 wild-type 400R or mutant 400C variant was assessed by MTT reduction assay after 48 h exposure to different concentrations of salsolinol following 12 h of incubation with cyclo(his-pro) (10 µmol/l). The 400C variant was less protected by cyclo(his-pro) against salsolinol-induced cell loss with an EC_50_ (95% confidence interval) of 406 (321 to 513) µmol/l compared to 763 (655 to 889) µmol/l (P<0.001) for 400R (n = 24–48; mean±s.e.m.; *P<0.05, ***P<0.001).

We further investigated whether the coding genetic variants of OCT2 with allelic frequencies ≥1% in at least one ethnic population (M165I, A270S, R400C and K432Q)[19] affect the transport of the endogenous OCT2 brain substrates. For cyclo(his-pro), the mutant 400C variant that occurs in African-Americans with an ethnic-specific allelic frequency of 1.5%[19] exhibited a significantly lower transport efficiency (V_max_/K_m_) and clearance (Cl_in_ ) compared to the wild-type 400R variant; whereas for salsolinol, transport kinetics were independent of the R400C genotype (**Fig. 5, A and B**). In contrast, transport rates for cyclo(his-pro) and salsolinol by the other OCT2 variants were not different from the wild-type control (**Tab. S1**). Consequently, HEK-293 cells transfected with the 400C genotype of OCT2 were less protected against salsolinol-induced cytotoxicity by cyclo(his-pro) pretreatment compared to the 400R genotype (**Fig. 5C**). This indicates that the shift in transport efficiency between cytoprotective and cytotoxic OCT2 substrates caused by the R400C substitution may increase susceptibility to cell death in the dopaminergic neurons and impact risk for PD. However, whether the R400C SNP is present to a greater degree in subjects with idiopathic PD has to be addressed in subsequent trials.

### Conclusion

We propose that the endogenous dopaminergic neuromodulators cyclo(his-pro) and salsolinol are key substrates of OCT2. Interestingly, both substrates are abundant in the dopaminergic brain region of the substantia nigra, where OCT2 shows its highest expression in CNS. Intracellular OCT2-mediated balance of salsolinol and cyclo(his-pro) appears to be crucial for maintenance of dopaminergic cell integrity. A rise in potentially cytotoxic salsolinol or a decline in protective cyclo(his-pro) may cause calcium-triggered apoptotic cell death, possibly contributing to the selective chronic nigral degeneration observed in PD.

In a broader perspective, our results provide the starting point for more detailed investigations of the function of OCT2 in the dopaminergic system and the potential association of genetically-determined alterations in OCT2 function with PD. Furthermore, the here presented technique provides principally new means to identify the leading endogenous substrates of any cell membrane transporter.

## Materials and Methods

### Chemicals

Cyclo(tyr-pro), pyroglutamyl-histidine, prolyl-alanine, and glycyl-proline were obtained from Bachem, salsolidine was from Carl Roth, and 1-methyl-3,4-dihydroisoquinoline and tetrahydropapaveroline were from Arcos Organics. All other chemicals were purchased from Sigma-Aldrich.

### Cloning and cell culture

The cDNAs encoding the organic cation transporters OCT1 (GenBank accession no. NM_003057), OCT2 (NM_003058), and EMT (NM_021977) were amplified by RT-PCR from human liver, kidney, and placenta total RNA respectively, and subcloned into the eukaryotic expression vector pcDNA3. The cDNA encoding the norepinephrine transporter (SLC6A2) was kindly provided by C. Pifl (University of Vienna). Genetic variants of OCT2 carrying a coding single nucleotide polymorphism (SNP) were generated from wild-type, pcDNA3-inserted cDNA by mutant oligonucleotide primers using the QuickChange Site-Directed Mutagenesis Kit (Stratagene). Human embryonic kidney cells (HEK-293) were stably transfected with the respective vectors and selected with geneticin as described.[8] Experiments were performed on cell clones exhibiting an equal mRNA expression level of the transporters, as determined by quantitative real-time PCR. HEK-293 cells, the human neuroblastoma cell line SH-SY5Y and the human glioblastoma cell line HTZ-146 (WHO grade IV) were cultured in DMEM (Dulbecco's Modified Eagle's Medium, Gibco) supplemented with 10% fetal bovine serum.

### Transport experiments

For uptake experiments, cells were grown on polystyrene dishes of 60 mm diameter to at least 80% confluence. Prior to addition of test compounds, culture medium was replaced by 3 ml HEPES-modified Krebs buffer (140.0 mmol/l NaCl, 5.0 mmol/l KCl, 2.0 mmol/l CaCl_2_, 1.0 mmol/l MgCl_2_, 10.0 mmol/l HEPES, and 5.0 mmol/l D-glucose, adjusted to pH 7.40), and cells were left to equilibrate for 60 min at 37°C. Uptake was terminated after indicated times by washing cells with ice-cold buffer. Cells were lysed with 1 ml of 4 mmol/l perchloric acid and lysates subjected to LC-MS-MS quantification. Cellular protein content was determined after solubilization of perchloric acid precipitates with 1 ml of 0.1 mmol/l NaOH using the bicinchoninic assay (BCA Protein Assay Kit, Pierce).

### Substrate identification by ESI-LC-MS-MS

HEK-293 cells expressing OCT2 or empty vector were incubated for 30 min with 100% fetal bovine serum (37°C, pH 7.4, Gibco) or rat whole brain homogenates (37°C, pH 7.4) that were prepared as described under ‘Determination of (cyclo)his-pro in rat brain’ below. After termination of uptake by washing with ice-cold HEPES-Krebs buffer and protein precipitation with 4 mmol/l perchloric acid or 100% acetonitrile in the case of brain homogenates, lysates were analyzed by LC-MS-MS operating in product scan positive electrospray ionisation mode (ESI) on a TSQ Quantum triple-quadrupole tandem mass spectrometer equipped with a thermostated (5°C) Surveyor autosampler and a thermostated (30°C) Surveyor HPLC system (Thermo Electron). Spray voltage was set at 4500 V, and capillary temperature was kept at 350°C. Nitrogen sheath gas and auxiliary gas pressure were 40 and 4 psi respectively. The collision gas was argon and collision-induced dissociation (CID) took place at 1.5 mTorr and 25 eV. 20 µl aliquots of the samples were injected onto a 5 µm Hilic normal phase column (100×3.0 mm; Waters Atlantis), and eluted isocratically at a flow rate of 0.25 ml/min (run time 8.0 min). The mobile phase consisted of 70% (v/v) methanol/0.1% formic acid and 30% (v/v) deionized water/0.1% formic acid. The resulting CID chromatograms reflect the total ion currents caused by the compound-specific ion transitions of the molecular ions [M+H]^+^ of a selected mass-to-charge ratio (m/z). 1-Methyl-4-phenyl-pyridinium iodide (MPP^+^, 500 ng/ml) was applied as internal standard (IS) and the m/z 170→128 transition was simultaneously recorded by single reaction monitoring (SRM) at 36 eV collision energy. The respective CID chromatograms obtained in control and OCT2 uptake experiments were corrected for differences in retention time of the IS (by shifting in x-direction) and for differences in peak area of the IS and in protein content of the cells (by shifting in y-direction). The difference chromatogram was generated from the modified CID chromatograms by running the product scan filter algorithm for the selected parent mass m/z. The ‘Background subtraction’ command in Xcalibur software version 1.4 (Thermo Electron) was applied to generate the difference chromatograms. For each parent mass, the CID scan file obtained with control cells was subtracted from the CID scan file obtained with OCT2 cells. Offsets in retention time of IS were incorporated in the model by specifying an alignment offset (in min), and offsets in IS area and protein content by specifying a proportional scaling factor according to SF = (IS_OCT2_×P_OCT2_)/(IS_control_×P_control_), where IS_OCT2_, IS_control_: peak area of SRM chromatograms of IS in OCT2 and control experiments, and P_OCT2_, P_control_: protein concentrations of cell lysates (in mg/ml) from OCT2 and control experiments. The difference spectrum corresponding to a specific difference chromatogram represents the characteristic fragment ions of a compound that is specifically transported by OCT2 and allows for most species an unambiguous assignment of the empirical formula and the chemical structure using mass spectral libraries (NIST MS Search algorithm; MS-Manager, ACD-Labs). Product-ion scanning analysis was repeated n-times for molecular ions of m/z = (100+n), n = 0,1,2…251 at a scan width of 0.5 each.

### Quantification by LC-MS-MS

Chromatographic separations were performed on Hypercarb (5 µm, 50×2.1 mm; Thermo Electron), Aquasil C18 (3 µm, 100×4.6 mm; Thermo Electron), or Hilic columns (5 µm, 100×3.0 mm; Waters Atlantis) by isocratic or gradient elution using 0.1% aqueous formic acid (A) and acetonitrile or methanol (B) as solvent system. For quantification, SRM transitions of a precursor ion [M+H]^+^ into a specific fragment ion (peak area ratios relative to IS) were recorded in positive ESI mode at 1.0–1.5 mTorr collision pressure and 10–40 eV collision energy. External calibration curves were obtained from serial dilutions of the analyte in 4 mmol/l HClO_4_ by linear or logistic regression. Detection limit was at least 0.001 nmol/mg protein. Compound-specific SRM transitions analyzed were: cyclo(his-pro): m/z 235→110; cyclo(tyr-pro): 261→136; pyroglutamyl-histidine: 267→110; pyroglutamic acid: 130→86; salsolinol: 180→117; N-methylsalsolinol: 194→151; salsolidine: 208→191; tetrahydropapaveroline: 289→123; proline: 116→70; prolineamide: 115→70; hydroxyproline: 132→86; glycylproline: 173→116; (1,2,3,4-)tetrahydroisoquinoline: 134→115; tetraethylammonium: 130→86; 1-methyl-3,4-dihydroisoquinoline: 146→103; tyramine: 138→121; prolylalanine: 187→70; TRH: 363→249; histamine: 112→68; carnosine: 227→110; tryptamine: 161→144; melatonine: 233→174; norharmane: 169→89; 6-methoxy-1,2,3,4-tetrahydro-β-carboline: 203→174.

### Extraction and quantification of mRNA

Multiple brain regions from a 63-year-old normal male were resected at autopsy at the University Hospital of Cologne (16 h postmortem), homogenized, and total RNA extracted by acid guanidinium thiocyanate-phenol-chloroform extraction.[20] Human total RNA samples (BD Biosciences, Clontech) of kidney, liver, pancreas, small intestine, colon, heart, skeletal muscle, spleen, lung, mammary gland, ovary, prostate, testis, and substantia nigra pars compacta were pooled from normal tissues of 10–30 Caucasian individuals. Total RNA of peripheral blood mononuclear cells (PBMCs, pooled from 5 healthy individuals) and cell cultures were extracted by the Roche High Pure RNA Isolation Kit. RNAs were reversed transcribed into cDNA by using random hexamers and Superscript II Plus RNase H^−^ Reverse Transcriptase (Invitrogen). Expression of SLC22A2 mRNA and in some experiments of SLC22A1, SLC22A3, NET (SLC6A2), and DAT (SLC6A3), and tyrosine hydroxylase (NM_199292.2) was determined from 10-ng samples of cDNA (as estimated from ethidium bromide spot tests) by quantitative real-time PCR (LightCycler, Roche Diagnostics) relative to the housekeeping gene glyceraldehyde-3-phosphate dehydrogenase (GAPDH) using TaqMan gene expression assays (Applied Biosystems). Assay IDs were as follows: Hs00427550_m1 (OCT1), Hs00533907_m1 (OCT2), Hs00222691_m1 (EMT), Hs01567436_m1 (NET), Hs00168988_m1 (DAT), Hs00165941_m1 (TH), Hs99999905_m1 (GAPDH). Relative quantifications were performed by pair-wise fixed reallocation randomization test[21] and corrected for amplification efficiency as evaluated from serial dilutions of cDNA. Repeated runs of the same samples (standards or unknowns) gave a maximal variability of 2–4% in CP values (interassay variation). Linearity of determinations was confirmed over the entire working range of mRNA expression (4–5 log values); failing expression was considered after more than 45 cycles of amplification without increase in fluorescence intensity.

### Determination of (cyclo)his-pro in rat brain

Complete brains were removed from heparinized and ether-sacrified Sprague-Dawley rats of either sex, weighting 200–250 g, dissected into its major anatomic divisions, washed in cold PBS, homogenized in liquid nitrogen, extracted in 10 ml 4 mmol/l HCLO_4_ by sonication, and centrifugated at 3,000 g at 4°C for 10 min. Rat kidney and liver were prepared in the same way. Concentrations of (cyclo)his-pro were measured in the supernatants by ESI-LC-MS-MS. Limit of quantification was 20 pmol/g of organ.

For uptake experiments (substrate search), homogenates of whole brain in liquid nitrogen were extracted with HEPES-modified Krebs buffer (pH 7.4, 10 ml per 3 g of homogenate) that was pre-heated to 95°C to denature enzymes, then shock frozen, and clarified by centrifugation at 20,000 g at 4°C for 30 min.

### Microdissection of human substantia nigra

Left and right sections of the pars compacta of substantia nigra (SNc) were separated at autopsy at the University Hospital of Cologne from 3 mm cross sectional midbrain slices of the normal brain of a 70-year-old woman (10 h postmortem) who died from a duodenal ulcer and had no clinical or pathological history of neuropsychiatric disease. The ventrolateral, ventromedial and dorsolateral subdivisions of the ventral and dorsal tiers of the left SNc were dissected out under microscopic control by use of a microslicer. The SNc regions were washed with cold PBS and homogenized in liquid nitrogen. One part was subjected to mRNA extraction and quantitative PCR and the other to determination of the intracellular concentrations of cyclo(his-pro), salsolinol and N-methyl-salsolinol by means of LC-MS/MS as described above. The lower limits of detection were 20 pmol/g tissue.

### Toxicity assays

Cell viability was assessed in HEK-293 cells, human dopaminergic neuroblastoma cells (SH-SY5Y) and human glioblastoma cells (HTZ-146) by the MTT test as described.[22] Briefly, cells were seeded onto 96-well plates (10,000 cells per well in 100 µl medium), and after 48–72 h of culture (80–90% confluence) incubated with salsolinol or glutamate for 48 h. In some cases, cells were incubated for 12 h (HEK-293) or 1 h (SH-SY5Y and HTZ-146) with 10 or 100 µmol/l (cyclo)his-pro prior to the addition of salsolinol or glutamate. In other experiments, MK-801, a selective antagonist of NMDA-receptor-gated calcium channels,[23] was concomitantly incubated with salsolinol or glutamate. For indication of metabolic activity, cells were treated with MTT reagent (15 µl of 1 mg/ml stock solution per well) for 1 h, and after drying of culture plates for 1 h at 37°C, the resulting formazan dye was extracted with isopropanol/HCl (100 µl 0.04 mol/l per well) and absorbance was determined spectrophotometrically at 568 nm. Results were in agreement with the data obtained by the tryptan blue exclusion method (data not shown).

### Kinetic and statistical analysis

Uptake velocity versus substrate concentration revealed saturation kinetics and was fitted by the Michaelis-Menten equation: V_0_ = V_max_·[S]/(K_m_+[S]), where V_0_ and V_max_ represent initial and maximal transport velocity respectively (nmol/mg protein/min), [S] initial substrate concentration (µmol/l), K_m_ substrate concentration at half maximal transport velocity (µmol/l), and V_max_/K_m_ efficiency of transport. Uptake versus time data were fitted by linear one-compartment kinetics: c_in_(t) = c_in,∞_·(1−e^−kex·t^), where c_in_(t) represents intracellular free substrate concentration after time t (pmol/mg protein), c_in,∞_ extrapolated maximal intracellular free substrate concentration (pmol/mg protein), and k_ex_ efflux rate constant (1/min). Influx clearance was calculated as CL_in_ = c_in,∞_/c_ex_·k_ex_ (µl/mg protein/min), with c_ex_ extracellular free substrate concentration (pmol/µl). Statistical significance of differences between two treatments was evaluated by the unpaired two-tailed t-test or Mann-Whitney U-test, differences between more than two treatments by one-way ANOVA or Kruskal-Wallis test, as applicable. P<0.05 was considered statistically significant.

## Supporting Information

Table S1 Impact of frequent OCT2 variants on transport kinetics of its endogenous key substrates
(0.04 MB DOC)

Figure S1 Identification of cyclo(his-pro) and salsolinol as selective endogenous substrates of the organic cation transporter OCT2 in the brain. Depicted are difference chromatograms and corresponding spectra of ESI-MS-MS fragmentation of the molecular ions [M+H]^+^ at m/z 180 (salsolinol) (A) and 235 (cyclo(his-pro)) (B) derived from acetonitrile lysates of OCT2 transfected HEK-293 cells versus empty vector transfected HEK-293 cells after 30 min of incubation with heat-inactivated rat whole brain homogenates, following 60 min preincubation in buffer solution. Specific fragment masses of the parent ions are indicated in red.
(1.62 MB TIF)

Figure S2 Efficiency of specific OCT2-mediated transport in stably transfected HEK-293 cells after 1 min of incubation (n = 3, mean±s.e.m.). Cyclo(his-pro) and salsolinol exhibited higher transport efficiencies (V_max_/K_m_) than tyramine and histamine (P<0.001): V_max_/K_m_(CHP) = 41, V_max_/K_m_(Sal) = 21, V_max_/K_m_(Tyr) = 9, and V_max_/K_m_(His) = 3 µl/mg protein/min. The transport efficiency for cyclo(his-pro) differed not significantly from the value for the tetraethylammonium (V_max_/K_m_ = 45 µl/mg protein/min; P = 0.863), which represents an effective, but unspecific model substrate of multiple transporters.
(0.03 MB TIF)

Figure S3 The pH influenced specific uptake of cyclo(his-pro) and salsolinol (10 µmol/l, 3 min incubation) in OCT2-transfected HEK-293 cells, indicating electrogenic transport that requires net positive charge. Cyclo(his-pro) and salsolinol are weak bases (pK_S_ = 6.5–7.5); thus acidic pH increases and alkaline pH decreases the proportion of protonated species (n = 3, mean±s.e.m.).
(0.90 MB TIF)

Figure S4 Specific uptake of cyclo(his-pro) and salsolinol (10 µmol/l, 3 min incubation) in OCT2-transfected HEK-293 cells was independent of extracellular sodium concentration. Sodium chloride was isoosmotically replaced with lithium chloride; 50 nmol/l NaCl abolished the transmembraneous sodium gradient (n = 3, mean±s.e.m.).
(0.91 MB TIF)

Figure S5 Comparison of specific uptake of cyclo(his-pro), salsolinol, and tyramine (10 µmol/l, 5 min incubation) between OCT2 and norepinephrine transporter (NET) transfected HEK-293 cells revealed that cyclo(his-pro) and salsolinol are no substrates of NET, while tyramine (positive control), in agreement with previous findings,[1] was an excellent NET substrate (n = 3, mean±s.e.m.). 1. Burnette WB, Bailey MD, Kukoyi S, Blakely RD, Trowbridge CG, et al. (1996) Human norepinephrine transporter kinetics using rotating disk electrode voltammetry. Anal Chem 68: 2932–2938.
(0.90 MB TIF)

Figure S6 Quantitative real-time expression of OCT2 mRNA in peripheral tissues (TaqMan Assay) normalized to GAPDH. Expression is given relative to pooled substantia nigra ( = 100%). Samples of normal tissue were pooled from at least five individuals; n.e. indicates no expression of OCT2, PBMC: peripheral blood mononuclear cells.
(0.88 MB TIF)
