# Supplementary material for: Identification of the Endogenous Key Substrates of the Human Organic Cation Transporter OCT2 and Their Implication in Function of Dopaminergic Neurons
Source: PLoS One. 2007 Apr 25;2(4):e385. doi: 10.1371/journal.pone.0000385 (PMC1851987; doi:10.1371/journal.pone.0000385)
Supplement: Table S1 — Impact of frequent OCT2 variants on transport kinetics of its endogenous key substrates (0.04 MB DOC) [file pone.0000385.s001.doc]

**Tab. S1.** Impact of frequent OCT2 variants on transport kinetics of its endogenous key substrates

|  | **Cyclo(his-pro)** | | | **Salsolinol** | | |
| --- | --- | --- | --- | --- | --- | --- |
| **OCT2 variants**  **(amino acid substitution)** | **V_max_**  **(nmol/mg/min)** | **K_m_**  **(µmol/l)** | **Cl_in_**  **µl/mg/min** | **V_max_**  **(nmol/mg/min)** | **K_m_**  **(µmol/l)** | **Cl_in_**  **µl/mg/min** |
| wild-type | 3.0 (0.2) | 74 (12) | 25.0 (4.2) | 2.7 (0.2) | 130 (24) | 18.5 (2.6) |
| M165I | 2.9 (0.3) | 78 (15) | 24.8 (3.7) | 2.5 (0.2) | 127 (22) | 19.3 (2.4) |
| A270S | 3.2 (0.2) | 75 (11) | 25.5 (3.3) | 2.7 (0.3) | 132 (26) | 18.1 (2.3) |
| R400C | 1.0 (0.1)^#^ | 51 (6)^#^ | 17.6 (1.5)^#^ | 2.6 (0.1) | 123 (13) | 20.9 (3.0) |
| K432Q | 3.1 (0.2) | 78 (12) | 26.0 (4.3) | 2.8 (0.2) | 129 (20) | 18.7 (2.1) |

Provided are maximal transport rate V_max_, and Michaelis-Menten constant K_m_ of specific uptake of cyclo(his-pro) or salsolinol into HEK-293 cells transfected with wild-type or genetic variants (SNPs) of OCT2 after 1 min of loading (n = 3, mean ± s.e.m) as well as influx clearance Cl_in_ of specific uptake after incubation with 10 µmol/l of cyclo(his-pro) or salsolinol (n = 3, mean ± s.e.m). ^#^P<0.01 indicates significant differences between wild-type and variant OCT2 as assessed by the unpaired two-tailed t-test.
