# Supplementary figures and images for: Identification of the Endogenous Key Substrates of the Human Organic Cation Transporter OCT2 and Their Implication in Function of Dopaminergic Neurons
Source: PLoS One. 2007 Apr 25;2(4):e385. doi: 10.1371/journal.pone.0000385 (PMC1851987; doi:10.1371/journal.pone.0000385)

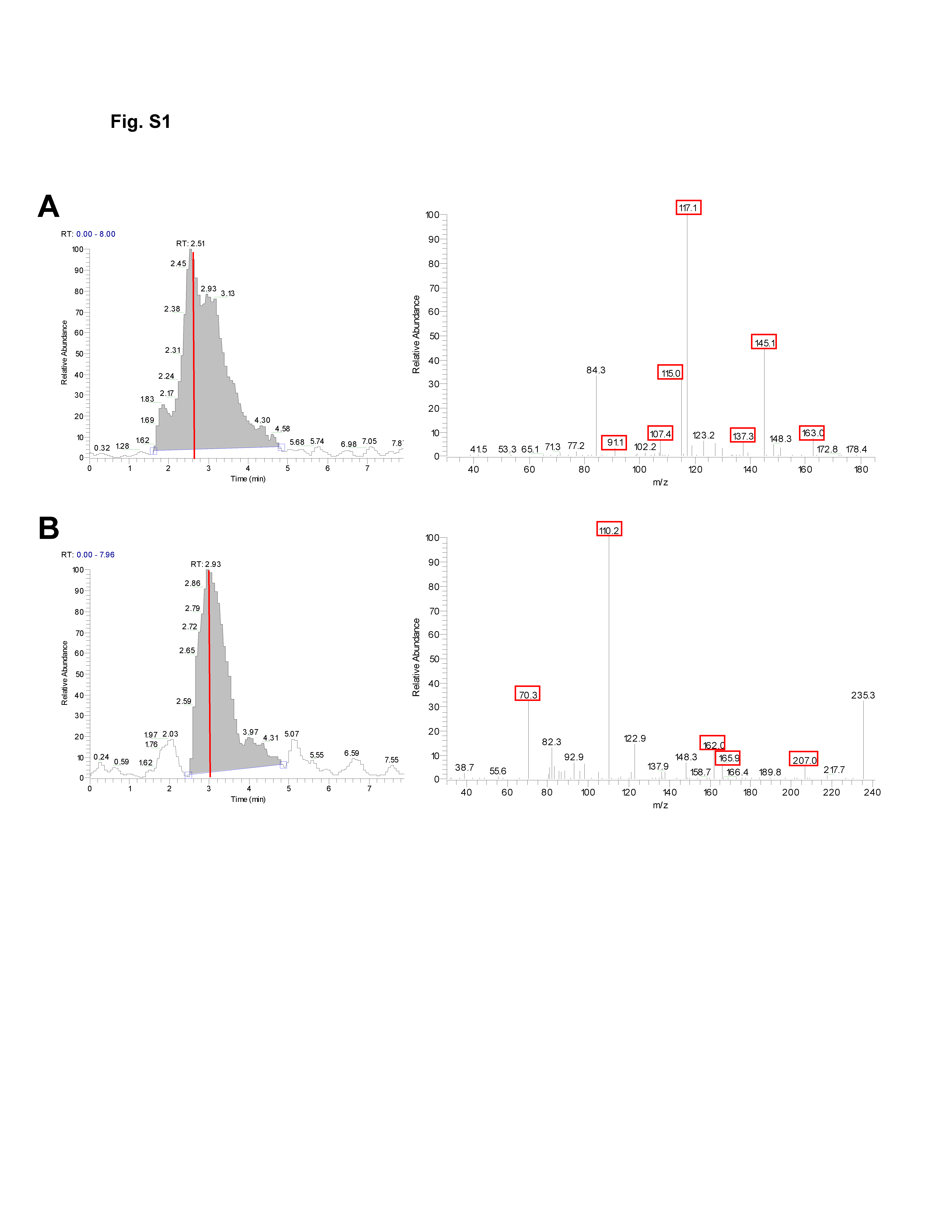

Supplement: Figure S1 — Identification of cyclo(his-pro) and salsolinol as selective endogenous substrates of the organic cation transporter OCT2 in the brain. Depicted are difference chromatograms and corresponding spectra of ESI-MS-MS fragmentation of the molecular ions [M+H]+ at m/z 180 (salsolinol) (A) and 235 (cyclo(his-pro)) (B) derived from acetonitrile lysates of OCT2 transfected HEK-293 cells versus empty vector transfected HEK-293 cells after 30 min of incubation with heat-inactivated rat whole brain homogenates, following 60 min preincubation in buffer solution. Specific fragment masses of the parent ions are indicated in red. (1.62 MB TIF) [file pone.0000385.s002.tif]

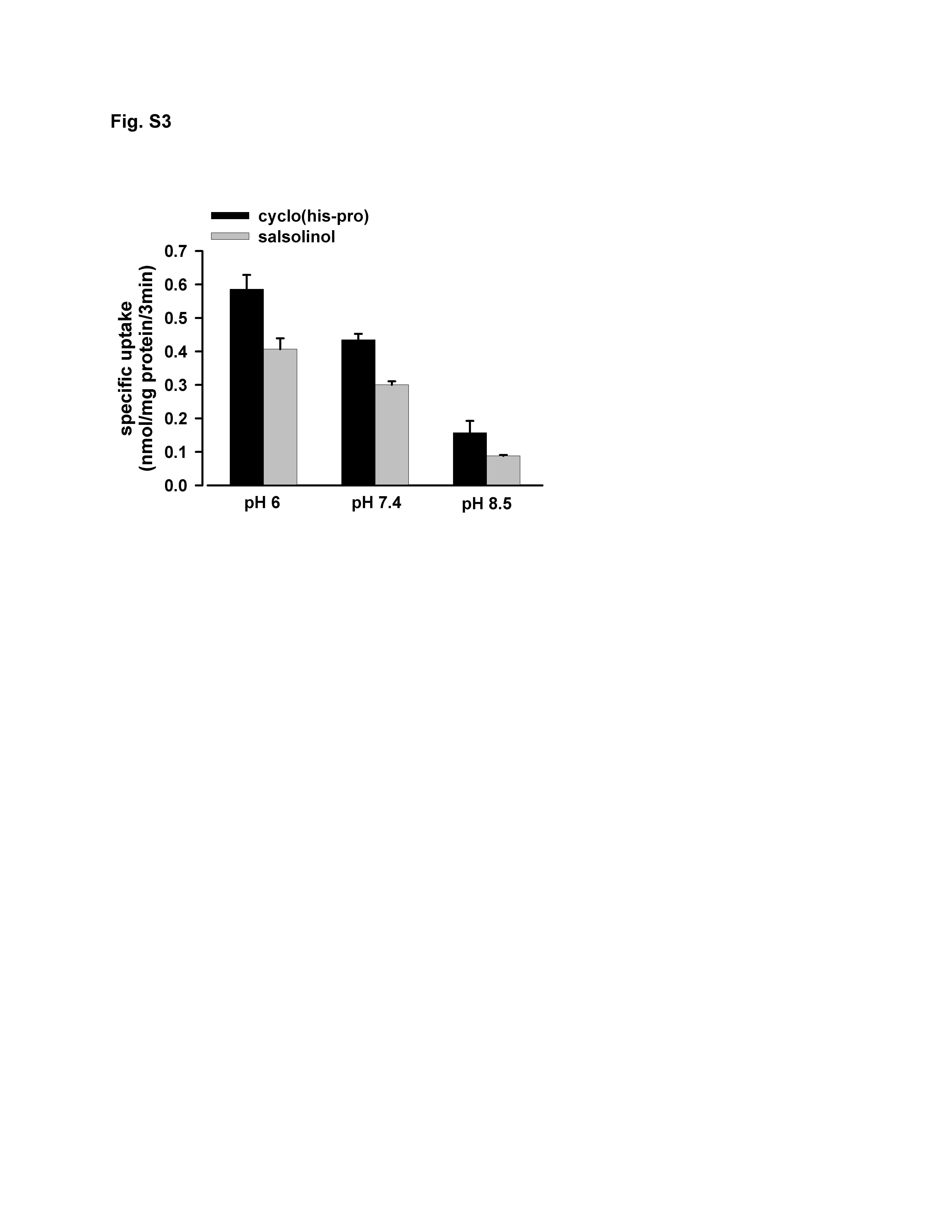

Supplement: Figure S3 — The pH influenced specific uptake of cyclo(his-pro) and salsolinol (10 µmol/l, 3 min incubation) in OCT2-transfected HEK-293 cells, indicating electrogenic transport that requires net positive charge. Cyclo(his-pro) and salsolinol are weak bases (pKS = 6.5–7.5); thus acidic pH increases and alkaline pH decreases the proportion of protonated species (n = 3, mean±s.e.m.). (0.90 MB TIF) [file pone.0000385.s004.tif]

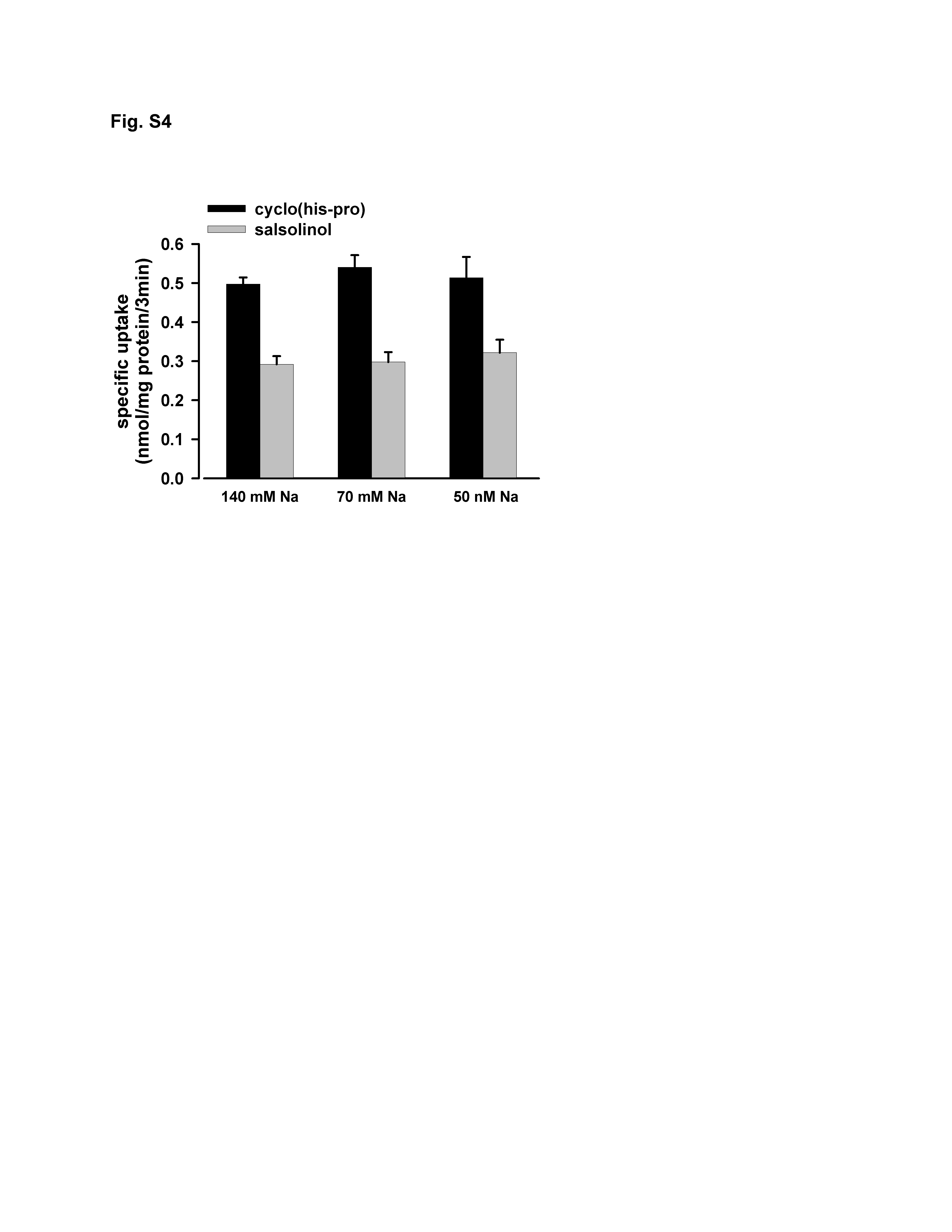

Supplement: Figure S4 — Specific uptake of cyclo(his-pro) and salsolinol (10 µmol/l, 3 min incubation) in OCT2-transfected HEK-293 cells was independent of extracellular sodium concentration. Sodium chloride was isoosmotically replaced with lithium chloride; 50 nmol/l NaCl abolished the transmembraneous sodium gradient (n = 3, mean±s.e.m.). (0.91 MB TIF) [file pone.0000385.s005.tif]

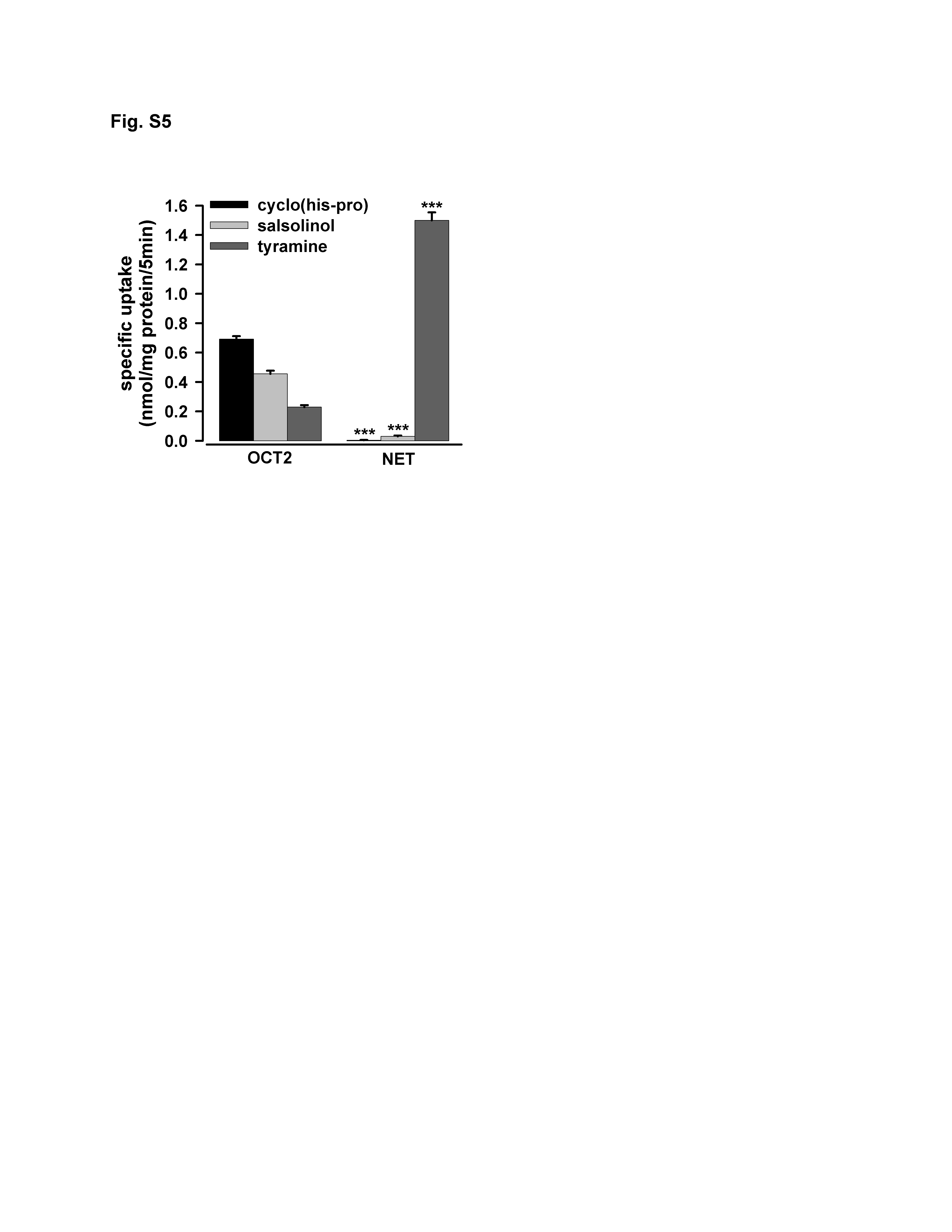

Supplement: Figure S5 — Comparison of specific uptake of cyclo(his-pro), salsolinol, and tyramine (10 µmol/l, 5 min incubation) between OCT2 and norepinephrine transporter (NET) transfected HEK-293 cells revealed that cyclo(his-pro) and salsolinol are no substrates of NET, while tyramine (positive control), in agreement with previous findings,[1] was an excellent NET substrate (n = 3, mean±s.e.m.). 1. Burnette WB, Bailey MD, Kukoyi S, Blakely RD, Trowbridge CG, et al. (1996) Human norepinephrine transporter kinetics using rotating disk electrode voltammetry. Anal Chem 68: 2932–2938. (0.90 MB TIF) [file pone.0000385.s006.tif]

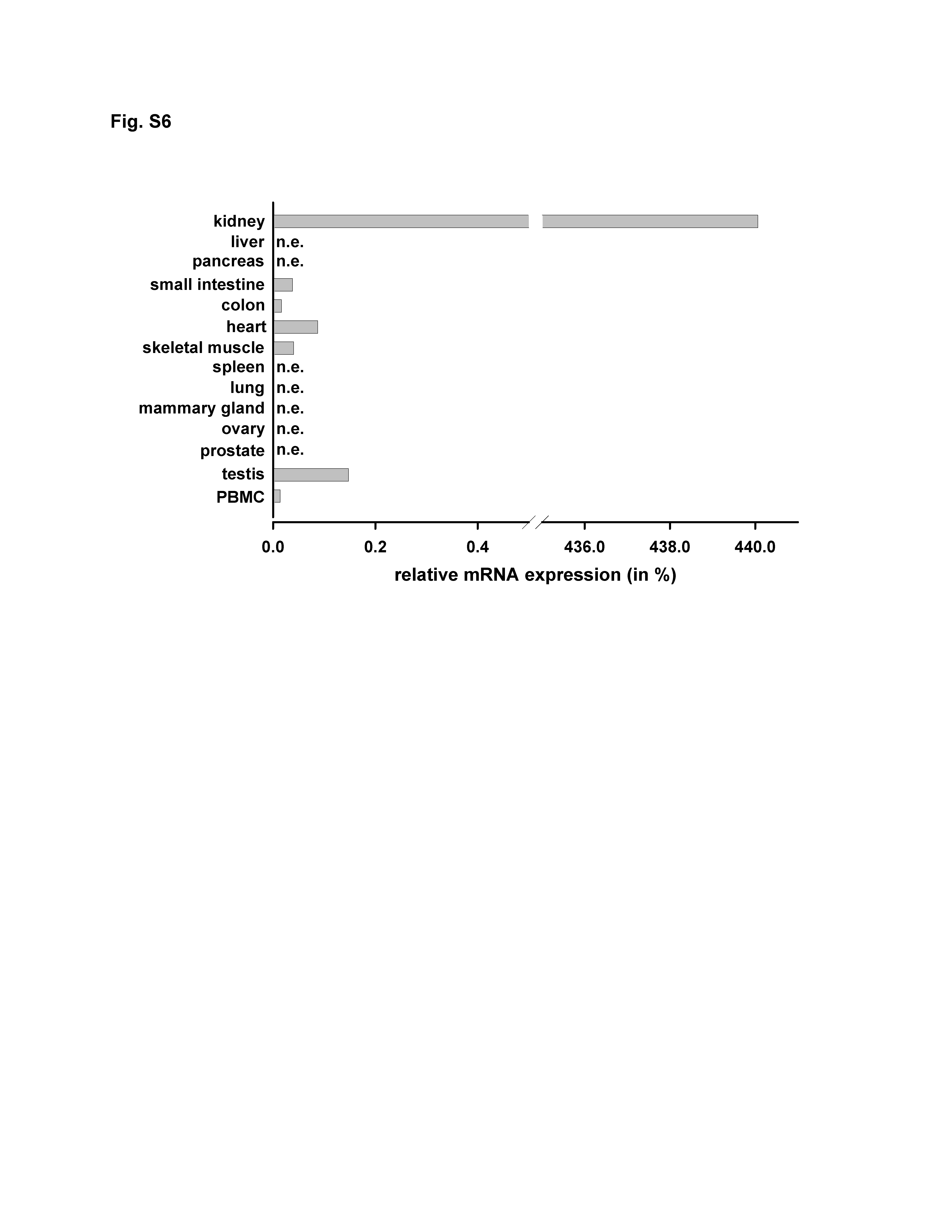

Supplement: Figure S6 — Quantitative real-time expression of OCT2 mRNA in peripheral tissues (TaqMan Assay) normalized to GAPDH. Expression is given relative to pooled substantia nigra ( = 100%). Samples of normal tissue were pooled from at least five individuals; n.e. indicates no expression of OCT2, PBMC: peripheral blood mononuclear cells. (0.88 MB TIF) [file pone.0000385.s007.tif]
